# Eight new species of *Batrisodes* Reitter from China (Coleoptera, Staphylinidae, Pselaphinae)

**DOI:** 10.3897/zookeys.694.13802

**Published:** 2017-08-29

**Authors:** Ri-Xin Jiang, Zi-Wei Yin

**Affiliations:** 1 Department of Biology, Shanghai Normal University, 100 Guilin Road, Shanghai, 200234, P. R. China

**Keywords:** *Batrisodes*, China, myrmecophilous, new species, Pselaphinae

## Abstract

Eight new species of the genus *Batrisodes* Reitter are described from continental China, seven of which were found in association with ants: *B. abdominalis* **sp. n.** and *B. tianmuensis* **sp. n.** with an *Ectomomyrmex* ant from Zhejiang; *B. grossus* **sp. n.** with an *Odontomachus* ant from Guangxi; *B. simianshanus* **sp. n.** with an *Aphaenogaster* ant from Chongqing; *B. qiului* **sp. n.** with a *Pheidole* ant, *B. xuhaoi* **sp. n.** with a *Lasius* ant, and *B. zhouchaoi* **sp. n.** with *Lasius* and *Nylanderia* ants from Sichuan. *Batrisodes zethus* **sp. n.** was collected from a leaf litter sample.

## Introduction

Eleven species of the genus *Batrisodes* Reitter are currently known to occur in China, scattered in Zhejiang, Hunan, Sichuan, Yunnan, Xizang (Tibet), Ningxia, and Taiwan (Besuchet 1981; Nomura 2007; Yin and Li 2013; Yin et al. 2011, 2015; Jiang and Yin 2016). This number is apparently at a low level in contrast to the 150 described species in the Holarctic region (Chandler 1997; Schülke and Smetana 2015).

Here, another eight new species are added to the Chinese fauna based on newly acquired material, seven of them were collected from ant colonies. This further demonstrates the high morphological disparity among members of *Batrisodes*, which, however, makes it more difficult to determine a synapomorphy for the genus.

## Materials and methods

All type material is housed in the Insect Collection of Shanghai Normal University, Shanghai, China (**SNUC**).

The collecting data of the material are quoted verbatim. The Chinese translation of each locality below provincial level is included in parentheses at the first appearance in the text. Each type specimen bears the following label: ‘HOLOTYPE (red) (or PARATYPE (yellow)), ♂ (or ♀), *Batrisodes* + specific name sp. n., det. Jiang and Yin 2017, SNUC’.

Dissected parts of dead beetles were preserved in Euparal on plastic slides that were placed on the same pin with the specimen. Habitus images were taken using a Canon 7D camera in conjunction with a Canon MP-E 65 mm f/2.8 1-5X Macro Lens, and a Canon MT-24EX Macro Twin Lite Flash was used as light source. Images of the morphological details were made using a Canon G9 camera mounted on an Olympus CX31 microscope under reflected or transmitted light. Zerene Stacker (version 1.04) was used for image stacking. All images were modified and grouped into plates in Adobe Photoshop CS5 Extended.

### The following abbreviations are applied

**AL** length of the dorsally visible part of the abdomen (posterior to elytra) along the midline;

**AnL** length of the antenna;

**AW** maximum width of the abdomen;

**EL** length of the elytra along the suture;

**EW** maximum width of the elytra;

**HL** length of the head from the anterior clypeal margin to the occipital constriction;

**HW** width of the head across eyes;

**PL** length of the pronotum along the midline;

**PW** maximum width of the pronotum.

Length of the body is a sum of HL + PL + EL + AL.

## Taxonomy

### Batrisodes abdominalis sp. n.

Taxon classificationAnimaliaColeopteraStaphylinidae

http://zoobank.org/E4EC7778-BD22-4790-BEE0-F5D505543627

Figure 1

#### Type material

(1 ex.). **Holotype: CHINA**: ♂, labeled ‘China: Zhejiang Prov., Linan County (临安县), West Tianmushan (西天目山), 06.v.2012, 1200 m, Wen-Xuan Bi leg.’ (SNUC).

#### Diagnosis of male.

The new species can be separated from other Chinese *Batrisodes* species by the following combination of characters: all antennomeres longer than wide; antennomeres X and XI each with a small denticle on the ventral side; mesofemur with a long protuberance near base, sternite V with a small spine at middle, and the slender, asymmetrical aedeagus with an elongate dorsal lobe.

#### Description.

**Figure 1.**
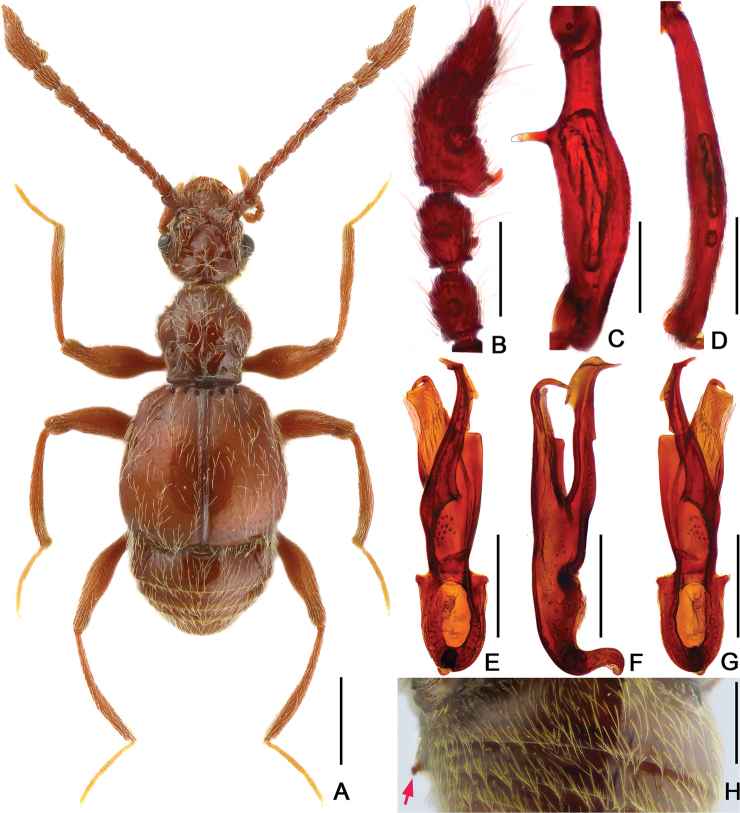
*Batrisodes abdominalis*, male. **A** Habitus **B** Antennal club **C** Mesofemur **D** Mesotibia **E–G** Aedeagus, in ventral (**E**), lateral (**F**), and dorsal (**G**) views **H** Abdominal segments V–VI, in lateral view. Scale bars: 0.5 mm (**A)**; 0.2 mm (**B–H**).

Male. (Fig. 1A), Body reddish brown, BL 2.62 mm. Head slightly wider than long, HL 0.51 mm, HW 0.57 mm, rectangular and covered with short hair, with large vertexal foveae, antennal tubercles prominent; area between moderately raised antennal tubercles concave and impunctate; clypeus slightly punctate, with round anterior margin; lateral longitudinal carinae slight, extending from level of eyes to head base, lacking median vertexal carina. Each eye composed of about 75 facets; Antennomeres II–XI longer than wide, IX–X (Fig. 1B) slightly enlarged, X with small denticle near basal 1/3; XI largest, nearly 2.5 times as long as X, with small denticle near base. Pronotum longer than wide, PL 0.63 mm, PW 0.55 mm, disc slightly convex, with small median antebasal foveae, median and lateral longitudinal sulci distinct; lateral antebasal fovea large and distinct; outer and inner basolateral foveae small but distinct. Elytra wider than long, without punctation and covered with sparse short hair, EL 0.89 mm, EW 0.97 mm, each elytron with three distinct basal foveae, discal striae shallow and unobvious. Mesofemora (Fig. 1C) with long distinct ventral spine near 1/3; mesotibiae (Fig. 1D) with short obtuse apical spine. Abdomen much wider than long, AL 0.59 mm, AW 0.81 mm; tergite IV longest, more than twice longer than next, with obvious oblique marginal carinae; sternite V (Fig. 1H) with small spine at middle. Aedeagus (Fig. 1E–G) slender and asymmetrical, median lobe simple with two elongate lobes. Length of aedeagus 0.61 mm.

Female. Unknown.

#### Distribution.

East China: Zhejiang.

#### Host ant.

*Ectomomyrmex* sp.

#### Biology.

The new species was collected from an ant colony nesting under a stone.

#### Etymology.

The specific epithet refers to the small spine on male sternite V.

### Batrisodes grossus sp. n.

Taxon classificationAnimaliaColeopteraStaphylinidae

http://zoobank.org/3C6404B4-F33D-47BA-8EAA-38340B8BA1A9

Figs 2 , 3 , 12

#### Type material

(5 exs). **Holotype: CHINA**: ♂, labeled ‘China: Guangxi, Jinxiu County (金秀县), Dayao Mountain (大瑶山), 16 km (十六公里), 24°08'11"N, 110°14'28"E, beech forest, rotten wood, colony of ant, 1100 m, 17.vii.2014, Zhong Peng leg.’ (SNUC). **Paratypes: CHINA**: 4 ♀♀, same label data as the holotype (SNUC).

#### Diagnosis of male.

The new species can be separated from other Chinese *Batrisodes* species by the following combination of characters: head dorsum, pronotum, and elytra roughly punctate, antennomeres III–V wider than VI–X, mesotrochanter, mesofemur, and mesotibia spinose, and slightly asymmetrical aedeagus expanded at the apex.

#### Description.

**Figure 2.**
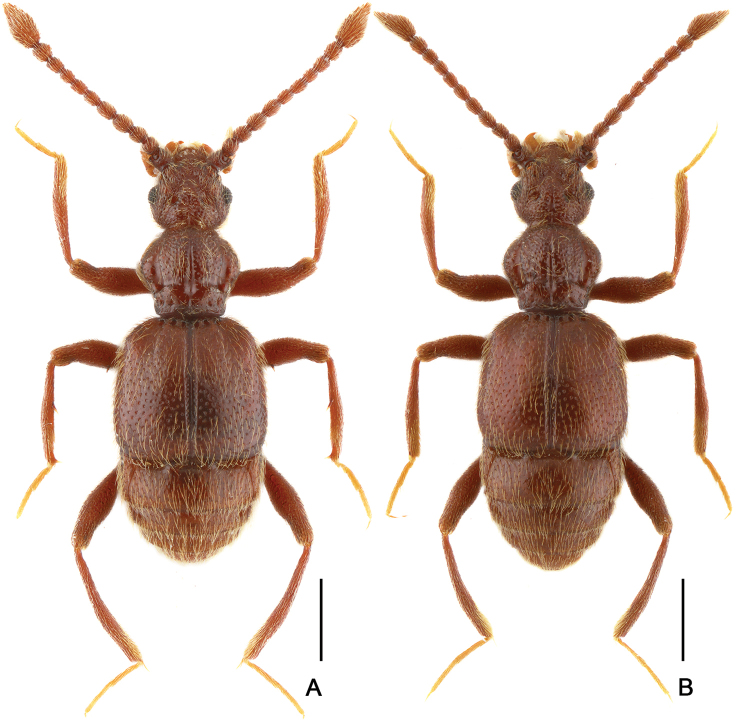
Dorsal habitus of *Batrisodes grossus*. **A** Male **B** Female. Scale bars: 0.5 mm.

**Figure 3.**
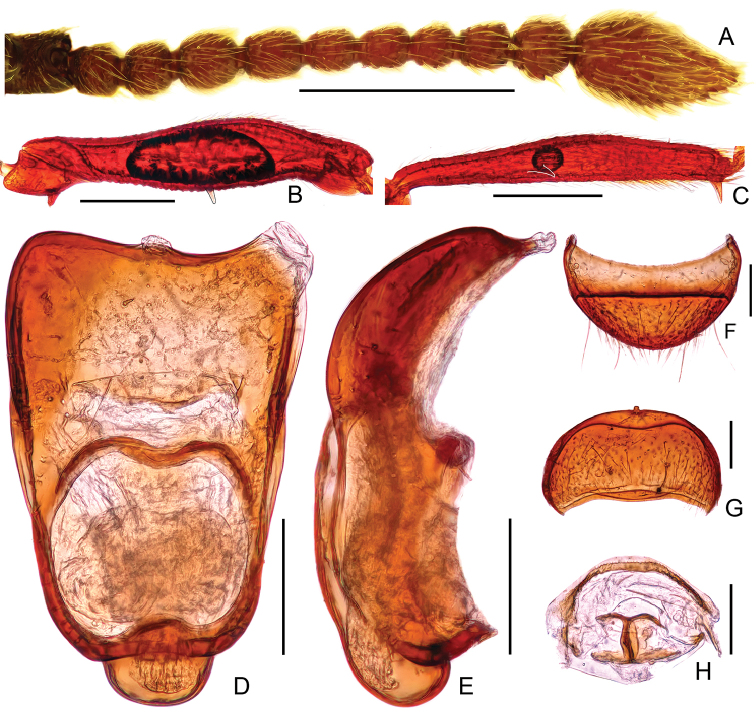
Diagnostic features of *Batrisodes grossus* (**A–E** Male **F–H** Female). **A** Antenna **B** Mesotrochanter and mesofemur **C** Mesotibia **D–E** Aedeagus, in ventral (**D**), and lateral (**E**) views **(F**) Tergite VIII **G** Sternite VIII **H** Genital complex. Scale bars: 0.2 mm (**A–C**); 0.1 mm (**D–H**).

Male. (Fig. 2A), Body reddish brown, BL 2.58 mm. Head approximately as long as wide, near trapezoidal, rough and covered with short hair, HL 0.46 mm, HW 0.50 mm, with large vertexal foveae, antennal tubercles prominent; area between moderately raised antennal tubercles concave; clypeus punctate, with round anterior margin; lateral longitudinal carinae slight, extending from level of eyes to head base, lacking median vertexal carina. Each eye composed of about 50 facets. Antennomeres II–X (Fig. 3A) moniliform, III–V slightly expanded, XI largest, nearly 2.5 times as long as X. Punctate pronotum slightly wider than long, PL 0.52 mm, PW 0.59 mm, disc slightly convex; with small median antebasal foveae, median and lateral longitudinal sulci distinct; lateral antebasal fovea large and distinct; outer and inner basolateral foveae small but distinct. Elytra as wide as long, with large uniform punctation and covered with long moderate-length hair, EL 0.87 mm, EW 0.87 mm; each elytron with three small but distinct basal foveae, discal striae shallow and unobvious. Mesotrochanter (Fig. 3B) with distinct triangular short spine; mesofemora (Fig. 3B) with thin but distinct ventral spine at middle; mesotibiae (Fig. 3C) with small ventral denticle near middle and an acute triangular apical spine. Abdomen wider than long, AL 0.73 mm, AW 0.85 mm; tergite IV longest, approximately 1.5 times as long as next, with strongly oblique marginal carinae. Length of aedeagus (Fig. 3D–E) 0.35 mm; median lobe simple, flattened, apical obviously expanded, nearly symmetrical.

Female (Fig. 2B). Similar to male, antennomere III–V normal; each eye composed of about 40 facets; legs lacking denticle and spine; tergite VIII (Fig. 3F) semicircular; sternite VIII (Fig. 3G) transverse; symmetrical genital complex (Fig. 3H) slightly sclerotized. Measurements of body parts: BL 2.45–2.53 mm, HL 0.45–0.46 mm, HW 0.50–0.51 mm, PL 0.49–0.52 mm, PW 0.58–0.60 mm, EL 0.81–0.84 mm, EW 0.87–0.88 mm, AL 0.70–0.71 mm, AW 0.83–0.84 mm.

#### Distribution.

Southwestern China: Yunnan.

#### Host ant.

*Odontomachus* sp.

#### Biology.

All adults were collected from an *Odontomachus* colony in a tree hole (Fig. 12).

#### Etymology.

The specific epithet refers to the roughly punctate body surface of the new species.

### Batrisodes qiului sp. n.

Taxon classificationAnimaliaColeopteraStaphylinidae

http://zoobank.org/7308BE97-B8E4-4701-AB67-91FA45B34D9D

Fig. 4

#### Type material

(1 ex.). **Holotype: CHINA**: ♂, labeled ‘China: Sichuan, Luzhou City, Gulin County (古蔺县), Honglong Lake (红龙湖), 28°07'17"N, 105°46'53"E, 1620 m, 30.iv.2017, ant nest in rotten wood, Lu Qiu leg.’ (SNUC)

#### Diagnosis of male.

The new species can be easily separated from other *Batrisodes* species in China by the following combination of characters: moniliform antennomeres, antennomeres XI with a large projection at the base, mesotrochanter with an abrupt projection at the ventral margin, mesofemur with a small spine at middle, and simple, slender aedeagus distinctly expanded at the apex.

#### Description.

**Figure 4.**
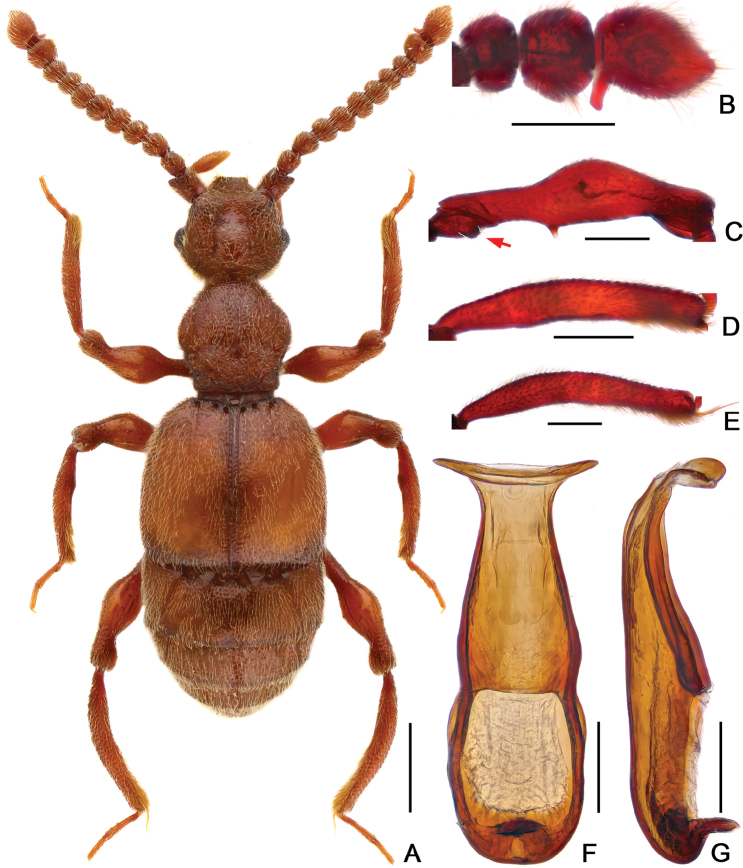
Diagnostic features of *Batrisodes qiului*, male. **A** Dorsal habitus **B** Antennal club **C** Mesotrochanter and mesofemur **D** Mesotibia **E** Metatibia **F–G** Aedeagus, in ventral (**F**), and lateral (**G**) views. Scale bars: 0.5 mm (**A**); 0.2 mm (**B–E**); 0.1 mm (**F–G**).

Male. (Fig. 4A), Body reddish brown, BL 3.05 mm. Head slight wider than long, near rectangular, rough and covered with short hair, HL 0.60 mm, HW 0.66 mm, with small but obvious vertexal foveae, antennal tubercles prominent; area between moderately raised antennal tubercles concave; clypeus slightly punctate, with round anterior margin; lateral longitudinal carinae unobvious, lacking median vertexal carina. Each eye composed of about 60 facets. Antennomeres II–XI moniliform, XI (Fig. 4B) largest, with distinct, apically-truncate basal denticle. Pronotum nearly as long as wide, PL 0.65 mm, PW 0.64 mm, disc slightly convex; with distinct median antebasal foveae, lateral longitudinal sulci present; lateral antebasal fovea distinct; outer and inner basolateral foveae small and not distinct. Elytra wider than long, EL 0.93 mm, EW 1.07 mm; each elytron with three small but distinct basal foveae, discal striae shallow and short. Mesotrochanter (Fig. 4C) with abrupt projection at ventral margin; mesofemora (Fig. 4C) with short but distinct ventral spine at basal 2/5; mesotibiae (Fig. 4D) slightly expanded at basal 1/3, with small apical spine; metatibiae (Fig. 4E) with long apical tuft of setae. Abdomen wider than long, AL 0.87 mm, AW 1.05 mm; tergite IV (first visible tergite) longest, approximately twice as long as next, with obvious oblique marginal carinae; tergite V–VI with obvious oblique marginal carinae. Aedeagus (Fig. 4F–G) slender, length 0.45 mm; median lobe simple, flattened and symmetrical, expanded at the apex.

Female. Unknown.

#### Distribution.

Southwestern China: Sichuan.

#### Host ant.

*Pheidole* sp.

#### Biology.

All adults were collected from an ant nest in rotten wood.

#### Etymology.

The specific epithet is dedicated to our friend Lu Qiu, who collected this new species and sent the material to us for study.

### Batrisodes simianshanus sp. n.

Taxon classificationAnimaliaColeopteraStaphylinidae

http://zoobank.org/67946A0E-7D8D-4B5E-AEAB-B9C55D77F2BB

Figs 5 , 6 , 13

#### Type material

(8 exs). **Holotype: CHINA**: ♂, labeled ‘China: Chongqing, Simian Shan N. R. (四面山自然保护区), Sunzigou (笋子沟), 28°41'47"N, 106°22'49"E, 751 m, 06.iii.2016, ant nest under rock, XU Hao & QIU Jianyue leg.’ (SNUC). **Paratypes: CHINA**: 1 ♂, 3 ♀♀, same label data as the holotype (SNUC); 2 ♂♂, 1 ♀, labeled ‘China, Chongqing City, Jiangjin District (江津区), Simianshan N. R. (四面山自然保护区), Motianling (摩天岭), ant nest under rock, 28°38'03"N, 106°22'59"E, 1220 m, 30.iv.2016, XU Hao & QIU Jianyue leg.’ (SNUC).

#### Diagnosis of male.

*Batrisodes simianshanus* can be separated from all other Chinese congeners by the following combination of characters: ocular canthi present, antennomere XI with a small, acute denticle at mesal margin, pronotum lacking outer and inner basolateral foveae, median antebasal foveae small, mesofemur with a distinct ventral protuberance near base, mesotibiae with small apical spine, and symmetrical, robust aedeagus with the endophallus bearing a pair of elongate lateral sclerites.

#### Description.

**Figure 5.**
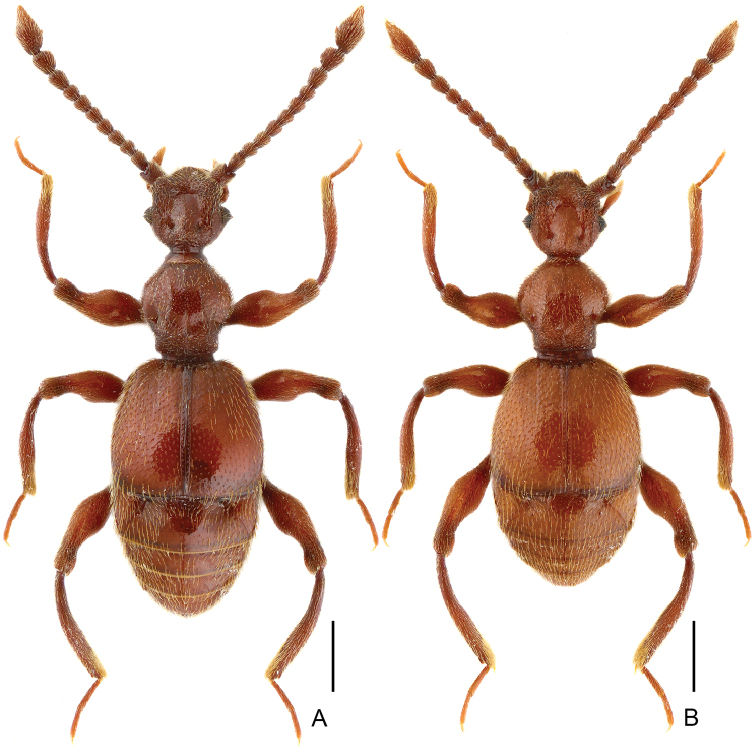
Dorsal habitus of *Batrisodes simianshanus*. **A** Male **B** Female. Scale bars: 0.5 mm.

**Figure 6.**
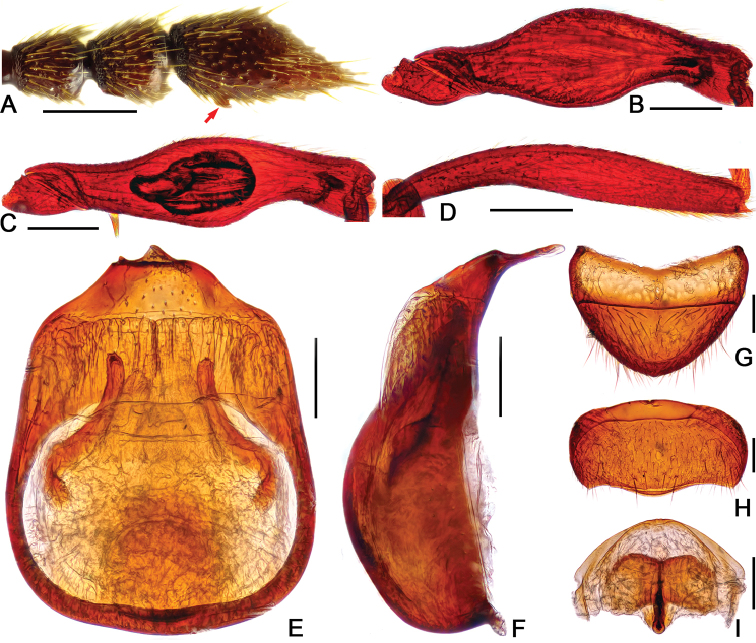
Diagnostic features of *Batrisodes simianshanus* (**A–F** Male **G–I** Female). **A** Antennal club (arrow indicates ventral denticle). **B** Protrochanter and profemur **C** Mesotrochanter and mesofemur **D** Mesotibia **E–F** Aedeagus, in ventral (**E**), and lateral (**F**) views **(G**) Tergite VIII (**H**) Sternite VIII (**I**) Genital complex. Scale bars: 0.2 mm (**A–D**); 0.1 mm (**E–I**).

Male. (Fig. 5A), Body reddish brown, BL 3.18–3.20 mm. Head wider than long, near rectangular, rough and covered with short hair HL 0.62–0.63 mm, HW 0.68 mm, with large vertexal foveae, antennal tubercles prominent; area between obviously raised antennal tubercles concave and without hair; clypeus slightly punctate, with round anterior margin; lateral longitudinal carinae slight, extending from level of eyes to head base. Each eye composed of about 60 facets and with one short ocular spine. Antennomeres II–X moniliform, IX–XI (Fig. 6A) slightly expanded, XI large and longest, with small denticle near base. Pronotum nearly as long as wide, PL 0.69–0.70 mm, PW 0.65–0.68 mm, disc slightly convex; with much small media antebasal foveae, median and lateral longitudinal sulci shallow and unclear; lateral antebasal fovea large and distinct, without outer and inner basolateral foveae. Elytra slight wider than long, EL 0.99–1.01 mm, EW 1.14–1.16 mm; each elytron with three small but distinct basal foveae, discal striae shallow and short. Profemora (Fig. 6B) expanded at middle, mesofemora (Fig. 6C) with thin but distinct ventral protuberance near base and expanded at middle; mesotibiae (Fig. 6D) with small and indistinct triangular apical spine. Abdomen wider than long, AL 0.85–0.89 mm, AW 1.04–1.09 mm; tergite IV longest, twice as long as next, with shallow but distinct oblique marginal carinae. Aedeagus (Fig. 6E–F) symmetrical and robust, median lobe simple, with pair of elongate lateral sclerites, length of aedeagus 0.44 mm.

Female (Fig. 5B). Similar to male, antennomere IX–XI less expanded, XI lacking denticle; each eye composed of about 50 facets; legs simple; tergite VIII (Fig. 6G) semicircular; sternite VIII (Fig. 6H) transverse; symmetrical genital complex (Fig. 6I) slightly sclerotized. Measurements of body parts: BL 3.01–3.04 mm, HL 0.62–0.63 mm, HW 0.63–0.64 mm, PL 0.66–0.68 mm, PW 0.65–0.66 mm, EL 0.98–0.99 mm, EW 1.10–1.12 mm, AL 0.74–0.75 mm, AW 1.04–1.09 mm.

#### Distribution.

Southwestern China: Chongqing.

#### Host ant.

*Aphaenogaster* sp.

#### Biology.

All adults were collected from ant colonies nested on the ground under stones (Fig. 13).

#### Etymology.

The specific epithet refers to the type locality of the new species, the Simianshan Nature Reserve.

### Batrisodes tianmuensis sp. n.

Taxon classificationAnimaliaColeopteraStaphylinidae

http://zoobank.org/EF6FF910-56BE-4AAE-A73C-D7411C002B58

Fig. 7

#### Type material.

(1 ex.). **Holotype: CHINA**: ♂, labeled ‘China: Zhejiang, Linan County, West Tianmushan, 13.iv.2017, 310 m, 30°18'54"N, 119°26'35"E, ant nest under rock, Song Xiaobin leg.’ (SNUC).

#### Diagnosis of male.

*Batrisodes tianmuensis* can be separated from all other Chinese congeners by the following combination of characters: antennomeres XI strongly concave at the ventral margin and with an acute basal projection, smooth pronotum lacking median antebasal foveae and inner and outer basolateral foveae, mesofemur with a small ventral spine at middle, mesotibia with a blunt apical protuberance, and aedeagus with an elongate, broad dorsal lobe suddenly pointed at the apex.

#### Description.

**Figure 7.**
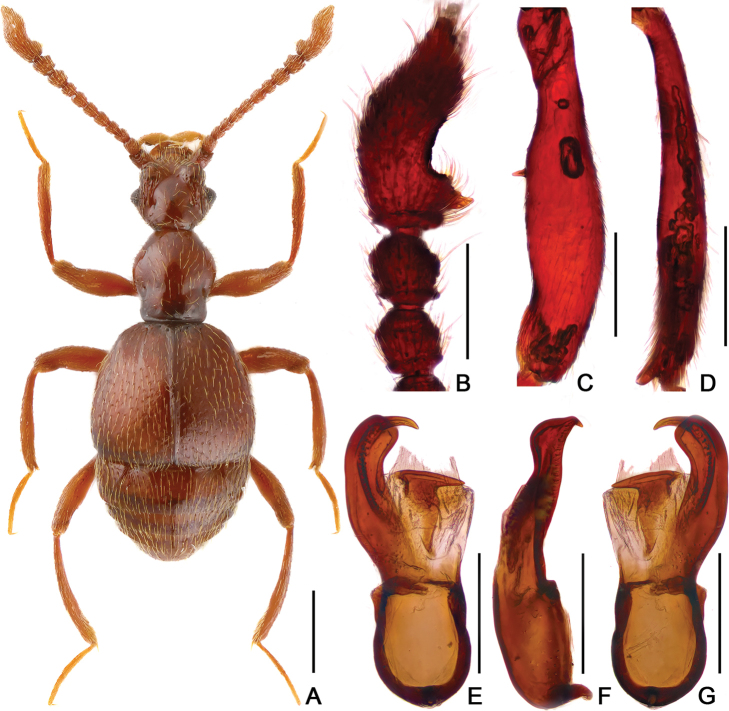
Diagnostic features of *Batrisodes tianmuensis*, male. **A** Dorsal habitus **B** Antennal club **C** Mesotrochanter and mesofemur **D** Mesotibia **E–G** Aedeagus, in ventral (**E**), lateral (**F**), and dorsal (**G**) views. Scale bars: 0.5 mm (**A**); 0.2 mm (**B–G**).

Male. (Fig. 7A), Body reddish brown, BL 2.52 mm. Head wider than long, rectangular and covered with sparse short hair, HL 0.51 mm, HW 0.57 mm, with small vertexal foveae; antennal tubercles prominent; area between moderately raised antennal tubercles concave and impunctate; clypeus slightly punctate, with round anterior margin; lateral longitudinal carinae slight, extending from level of eyes to head base, lacking median vertexal carina. Each eye composed of about 55 facets. Antennomeres II–X moniliform, XI (Fig. 7B) largest, approximately 3 times as long as X, strongly concave at mesal surface, with acute denticle near base. Pronotum slightly longer than wide, PL 0.55 mm, PW 0.52 mm, smooth with sparse short hair; disc slightly convex, without median antebasal foveae and median longitudinal sulci; lateral longitudinal sulci shallow, lateral antebasal fovea small and indistinct; lacking outer and inner basolateral foveae. Elytra wider than long, smooth and covered with sparse short hair, EL 0.87 mm, EW 0.99 mm; each elytron with three small basal foveae, discal striae shallow and indistinct. Mesofemora (Fig. 7C) with small thin ventral spine near middle; mesotibiae (Fig. 7D) with small triangular apical spine. Abdomen much wider than long, AL 0.62 mm, AW 0.95 mm; tergite IV longest, nearly 2.5 times as long as next, with shallow oblique marginal carinae. Aedeagus (Fig. 7E–G) strongly asymmetrical, median lobe with long, broad dorsal lobe pointed at apex; length of aedeagus 0.49 mm.

Female. Unknown.

#### Distribution.

East China: Zhejiang.

#### Host ant.

*Ectomomyrmex* sp.

#### Biology.

The new species was collected from an ant colony nesting under a stone.

#### Etymology.

The specific epithet refers to the type locality of the new species, the West Tianmu Mountain.

### Batrisodes xuhaoi sp. n.

Taxon classificationAnimaliaColeopteraStaphylinidae

http://zoobank.org/401C578A-AA7F-459E-80DA-AD99CB5B216A

Figs 8 , 9 , 14

#### Type material

(5 exs). **Holotype: CHINA**: ♂, labeled ‘China: Sichuan, Luding County (泸定县), Gonggashan N. R. (贡嘎山自然保护区), Hongshitan (红石滩), ant nest under rock, 29°48'10"N, 102°03'42"E, 2740 m, 24.vi.2016, XU Hao & QIU Jianyue leg.’ (SNUC). **Paratypes: CHINA**: 3 ♂♂, 1 ♀, same label data as the holotype (SNUC).

#### Diagnosis of male.

*Batrisodes xuhaoi* can be separated from all other Chinese congeners by the following combination of characters: stout general habitus, moniliform antennomeres, antennomere XI with a thick projection at base, profemur strongly expanded near middle, mesotrochanter expanded at ventral margin, mesofemur with a small ventral spine at middle, mesotibia with acute spines at middle and apex, and asymmetrical, elongate aedeagus broadened at the apex.

#### Description.

**Figure 8.**
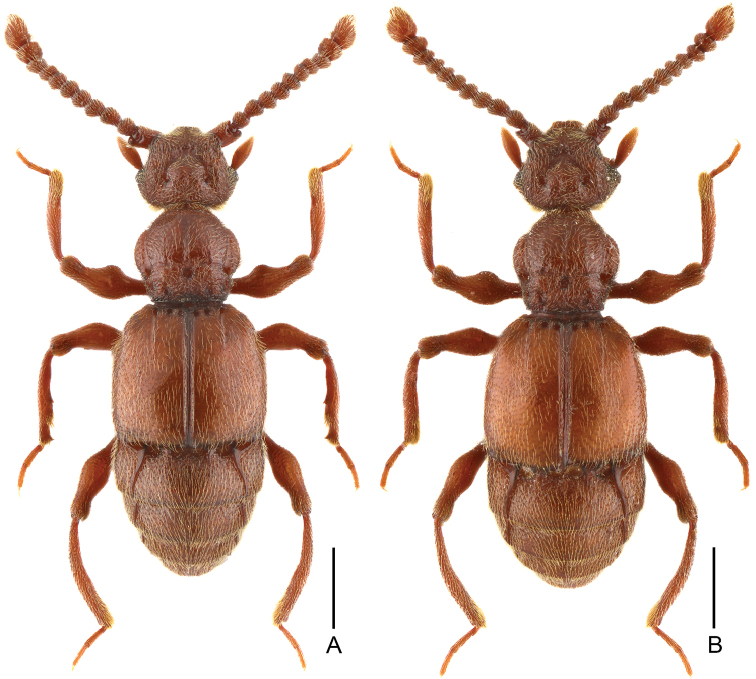
Dorsal habitus of *Batrisodes xuhaoi*. **A** Male **B** Female. Scale bars: 0.5 mm.

**Figure 9.**
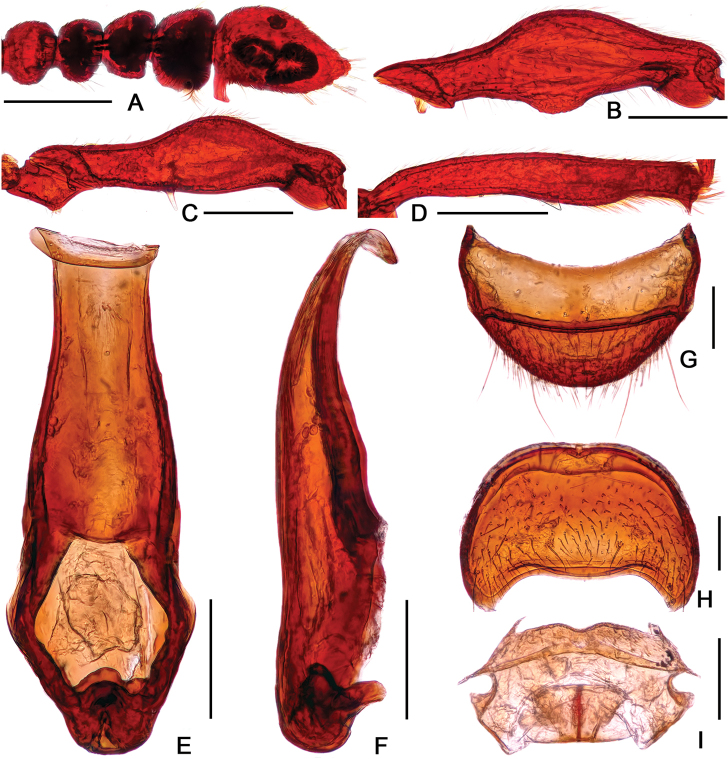
Diagnostic features of *Batrisodes xuhaoi* (**A–F** Male **G–I** Female). **A** Antennal club **B** Profemur **C** Mesotrochanter and mesofemur **D** Mesotibia **E–F** Aedeagus, in ventral (**E**), and lateral (**F**) views (**G**) Tergite VIII (**H**) Sternite VIII (**I**) Genital complex. Scale bars: 0.2 mm (**A–D**); 0.1 mm (**E–I**).

Male. (Fig. 8A), Body reddish brown, BL 2.76–2.80 mm. Head wider than long, near trapezoidal, rough and covered with short hair, HL 0.50–0.51 mm, HW 0.62–0.63 mm, with large vertexal foveae, antennal tubercles prominent; area between moderately raised antennal tubercles obviously concave; clypeus slightly punctate, with round anterior margin; lateral longitudinal carinae short and slight, extending from level of eyes to head base, lacking median vertexal carina. Each eye composed of about 50 facets. Antennomeres II–XI moniliform, XI (Fig. 9A) large, with distinct, apically-truncate basal denticle. Pronotum as long as wide, PL 0.63–0.64 mm, PW 0.63–0.64 mm, disc slightly convex; with distinct median antebasal foveae, median and lateral longitudinal sulci present; lateral antebasal fovea large and distinct; outer and inner basolateral foveae small but distinct. Elytra wider than long, EL 0.85–0.86 mm, EW 0.92–0.94 mm; each elytron with three small but distinct basal foveae, discal striae shallow and short. Profemora (Fig. 9B) strongly expanded at middle, mesofemora (Fig. 9C) with thin but distinct ventral spine near middle; mesotibiae (Fig. 9D) slightly swollen at apical 1/3, with small ventral denticle near middle and small triangular apical spine. Abdomen wider than long, AL 0.77–0.80 mm, AW 0.90–0.93 mm; tergite IV (first visible tergite) longest, nearly twice as long as next, with strongly oblique marginal carinae; tergite V–VI with obvious oblique marginal carinae. Aedeagus (Fig. 9E–F) nearly symmetrical, slender, length 0.44 mm, median lobe simple, flattened, broadened at apex.

Female (Fig. 8B). General habitus similar to male, antennomere XI lacking basal denticle; each eye composed of about 40 facets; legs lacking denticle and spine; tergite VIII (Fig. 9G) semicircular; sternite VIII (Fig. 9H) transverse; symmetrical genital complex (Fig. 9I) slightly sclerotized. Measurements of body parts: BL 2.82 mm, HL 0.51 mm, HW 0.64 mm, PL 0.65 mm, PW 0.64 mm, EL 0.88 mm, EW 0.96 mm, AL 0.78 mm, AW 0.93 mm.

#### Distribution.

Southwestern China: Sichuan.

#### Host ant.

*Lasius* sp.

#### Biology.

All adults were collected from ant colonies nesting on the ground under stones (Fig. 14).

#### Etymology.

The specific epithet is dedicated to Hao Xu, co-collector of the type series.

### Batrisodes zethus sp. n.

Taxon classificationAnimaliaColeopteraStaphylinidae

http://zoobank.org/79C51DBA-2BE7-4CC4-B4AB-7D94F5DB9080

Fig. 10

#### Type material

(1 ex.). **Holotype: CHINA**: ♂, labeled ‘China: Hunan, Liuyang City, Daweishan National Forest Park (大围山国家森林公园), 28°25'25"N, 114°07'06"E, 1391 m, 03. vi.2017, Jiang, Liu & Hu leg.’ (SNUC)

#### Diagnosis of male.

*Batrisodes zethus* can be separated from all other Chinese congeners by the following combination of characters: habitus stout, antennomere IX strongly protruding laterally, X oblique, XI with a setose longitudinal projection at the lateral surface, mesofemur with a distinct ventral projection at basal 1/3, mesotibia with small tubercles at middle and apex, aedeagus elongate, with numerous spine-like structures at middle of the ventral lobe.

#### Description.

**Figure 10.**
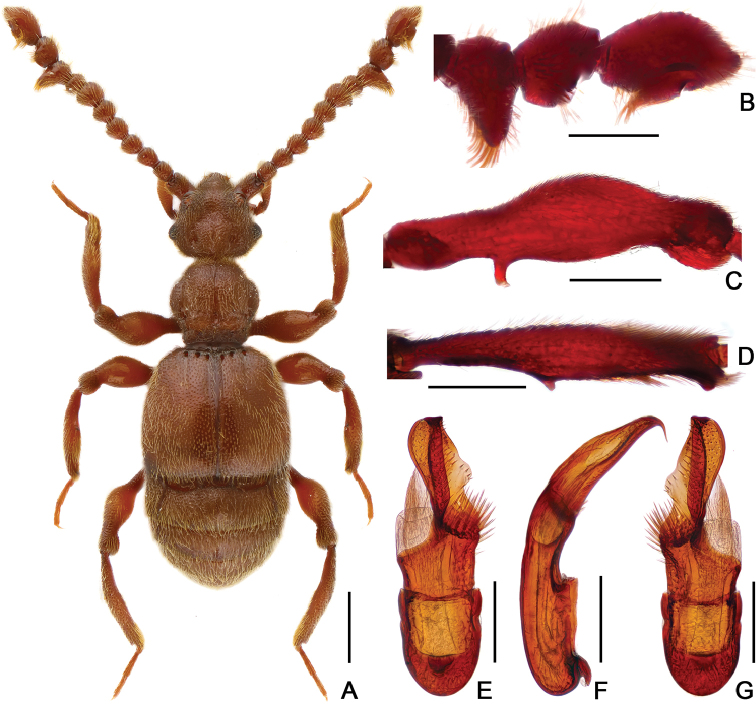
Diagnostic features of *Batrisodes zethus*, male. **A** Dorsal habitus **B** Antennal club **C** Mesotrochanter and mesofemur **D** Mesotibia **E–G** Aedeagus, in ventral (**E**), lateral (**F**), and dorsal (**G**) views. Scale bars: 0.5 mm (**A**); 0.2 mm (**B–G**).

Male. (Fig. 10A), Body reddish brown, BL 2.83 mm. Head wider than long, sub-triangular, roughly punctate and with short setae, HL 0.60 mm, HW 0.66 mm, with large vertexal foveae, antennal tubercles prominent, area between tubercles regularly depressed; clypeus finely punctate, with round anterior margin; lacking lateral longitudinal carinae and median vertexal carina. Each eye composed of about 60 facets. Antennomeres II–VIII moniliform, IX (Fig. 10B) strongly protruding at lateral margins and with long hair at apex, X oblique, XI with a distinct longitudinal projection near middle. Pronotum slightly wider than long, PL 0.58 mm, PW 0.62 mm, covered with fine, short hair, disc slightly convex; with small but , distinct mediobasal impression, lateral longitudinal sulci present; lateral antebasal fovea large and distinct; outer and inner basolateral foveae small. Elytra wider than long, uniformly punctate; EL 0.87 mm, EW 1.04 mm; each elytron with three large basal foveae, discal striae shallow, extending to half elytral length. Mesofemora (Fig. 10C) with thick, long projection at basal 1/3; mesotibiae (Fig. 10D) with small, blunt projection at middle and apex; Abdomen wider than long, AL 0.78 mm, AW 0.97 mm; tergite IV (first visible tergite) longest, nearly 1.5 times as long as next, with oblique marginal carinae; tergite V–VI each with oblique marginal carinae. Aedeagus (Fig. 10E–G) asymmetrical, median lobe with many spine-like structures at apical half.

#### Female.

Unknown.

#### Distribution.

Central China: Hunan.

#### Biology.

This new species was collected from leaf litter.

#### Etymology.

Zethus is the son of Zeus and Antiope.

### Batrisodes zhouchaoi sp. n.

Taxon classificationAnimaliaColeopteraStaphylinidae

http://zoobank.org/31133048-37CD-4B88-BBAE-76BFBA4D73B5

Fig. 11

#### Type material

(2 exs). **Holotype: CHINA**: ♂, labeled ‘China: Sichuan, Chengdu City (成都市), Dujiangyan City (都江堰市), Zipingpu Township (紫坪铺镇), Ling-yan-guan-yin-shan (灵岩观音山), 31.03°N, 103.61°E, 1180 m, 03.iv.2017, ant nest under rock, Zhou Chao & He Li leg.’ (SNUC). **Paratypes: CHINA**: 1 ♂, same collecting data as the holotype, except ‘ant colony under bark,’ (SNUC).

#### Diagnosis.

*Batrisodes zhouchaoi* can be readily separated from all other Chinese congeners by the following combination of characters: antennomere X strongly transverse, with a small denticle at mesal margin, XI with a small denticle base; pronotum distinctly winder than long; mesotrochanter with a blunt ventral projection, mesofemora with a long ventral protuberance at middle, mesotrochanter protuberant ventrally, metafemora expanded along the ventral margin; aedeagus strongly asymmetrical, with elongate, twisted ventral and dorsal lobes.

#### Description.

**Figure 11.**
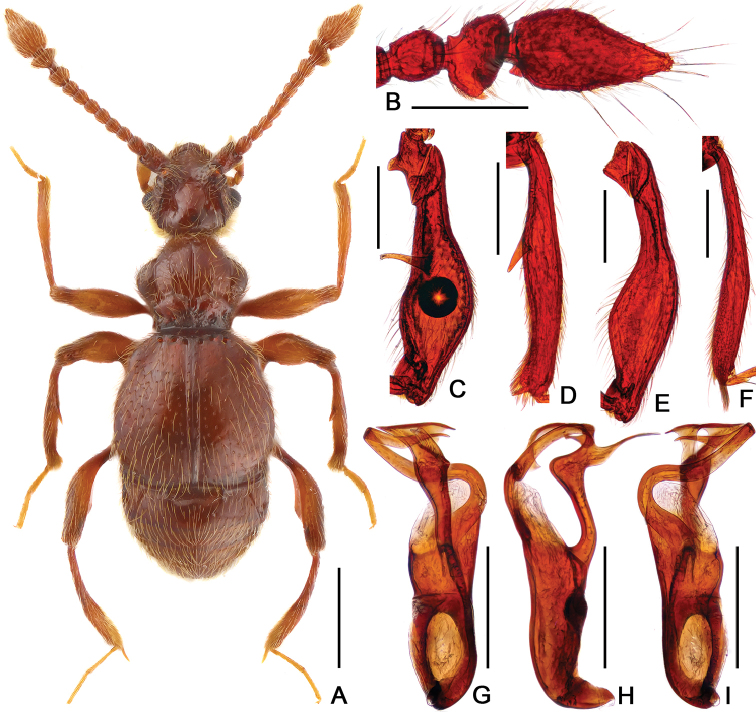
Diagnostic features of *Batrisodes zhouchaoi*, male. **A** Dorsal habitus **B** Antennal club **C** Mesotrochanter and mesofemur **D** Mesotibia **E** Metatrochanter and metafemur **F** Metatibia **G–I** Aedeagus, in ventral (**G**), lateral (**H**), and dorsal (**I**) views. Scale bars: 0.5 mm (**A**); 0.2 mm (**B–I**).

Male. (Fig. 11A), Body reddish brown, BL 2.18–2.19 mm. Head wider than long, near rectangular, and covered with short hair HL 0.45–0.46 mm, HW 0.50–0.51 mm, with large vertexal foveae, antennal tubercles prominent and punctate; area between obviously raised antennal tubercles concave and sparsely pubescent; clypeus slightly punctate, with round anterior margin; lateral longitudinal carinae extending from above eyes to occipital constriction. Each eye composed of about 55 facets. Antennomeres II–IX moniliform, X (Fig. 11B) much wider than long with small denticle near middle, XI (Fig. 11B) largest and with small denticle near base. Pronotum wider than long, PL 0.48–0.50 mm, PW 0.55–0.56 mm, disc slightly convex; with small antebasal foveae, median and lateral longitudinal sulci clear; lateral antebasal fovea large and distinct, outer and inner basolateral foveae distinct. Elytra slight wider than long, EL 0.74–0.75 mm, EW 0.77–0.78 mm; each elytron with three large basal foveae, discal striae shallow and short. Mesotrochanter (Fig. 11C) with blunt, short spine; mesofemora (Fig. 11C) expanded at apical 1/3 and with long and distinct ventral spine at middle; mesotibiae (Fig. 11D) with distinct ventral denticle spine near middle and a small triangular apical denticle; metafemora (Fig. 11E) expanded along ventral margin, metatibiae (Fig. 11F) with long apical tuft of setae. Abdomen wider than long, AL 0.48–0.51 mm, AW 0.72–0.74 mm; tergite IV longest, three times as long as next, with distinct oblique marginal carinae. Aedeagus (Fig. 11G–I) slender and asymmetrical, median lobe simple with two elongate and twisted lobe; length of aedeagus 0.48 mm.

Female. Unknown.

#### Distribution.

Southwestern China: Sichuan.

#### Host ant.

*Lasius* sp. and *Nylanderia* sp.

#### Biology.

All adults were collected from ant colonies nesting under stone or bark.

#### Etymology.

The new species is named after Chao Zhou, who collected this new species and sent us the material as a gift.

**Figure 12.**
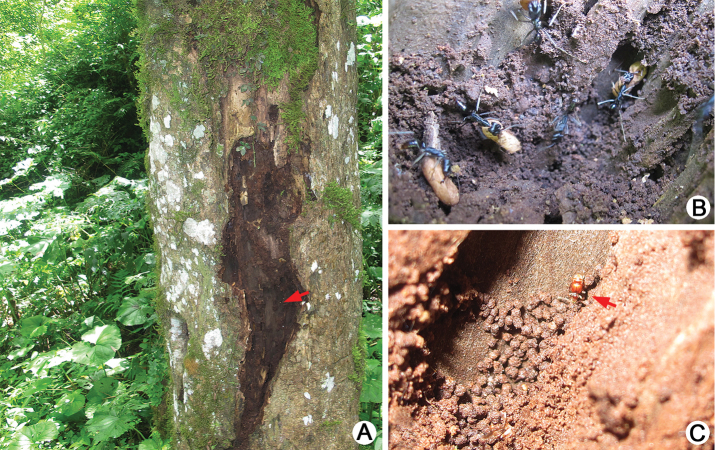
Habitat of *Batrisodes grossus*. **A** Colony of host ant under bark **B** A closer view of ant colony **C** A living *Batrisodes grossus* walking inside the nest.

**Figure 13.**
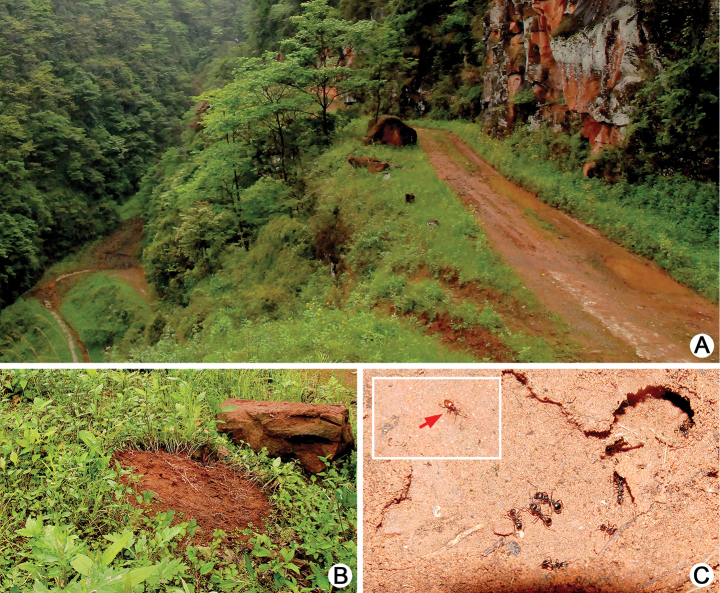
Habitat of *Batrisodes simianshanus*. **A** General environment **B** Ant nest exposed after the stone was turned over **C** A closer view of ant nest and a living *Batrisodes simianshanus* walking on the surface of ant nest.

**Figure 14.**
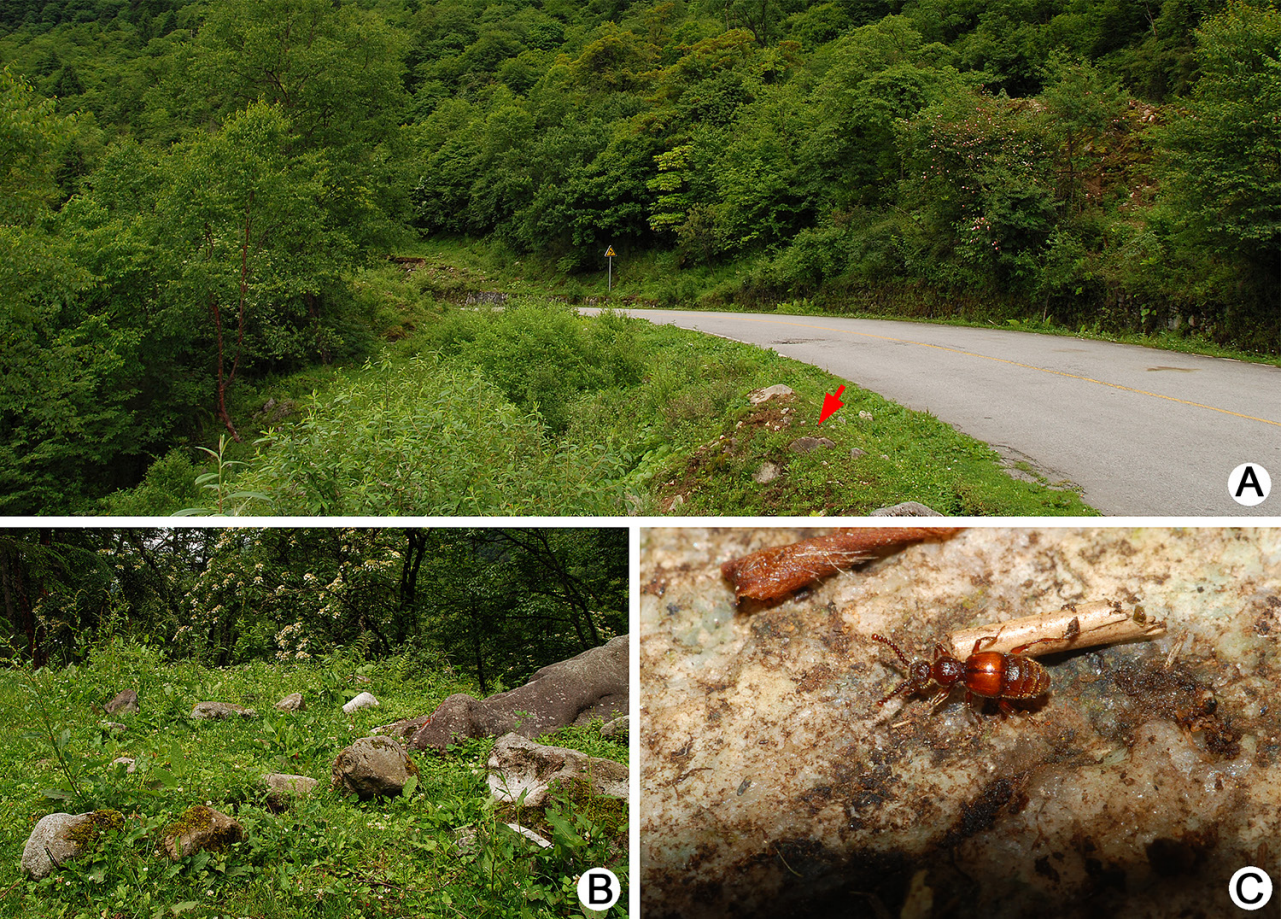
Habitat of *Batrisodes xuhaoi*. **A** General environment **B** A closer view of the habitat **C** A living *Batrisodes xuhaoi* walking on the underside of the stone.

## Supplementary Material

XML Treatment for Batrisodes abdominalis

XML Treatment for Batrisodes grossus

XML Treatment for Batrisodes qiului

XML Treatment for Batrisodes simianshanus

XML Treatment for Batrisodes tianmuensis

XML Treatment for Batrisodes xuhaoi

XML Treatment for Batrisodes zethus

XML Treatment for Batrisodes zhouchaoi
